# Dose-Effect of Zearalenone on the Localization and Expression of Growth Hormone, Growth Hormone Receptor, and Heat Shock Protein 70 in the Ovaries of Post-weaning Gilts

**DOI:** 10.3389/fvets.2021.629006

**Published:** 2021-02-04

**Authors:** Tingting Song, Xiufeng Liu, Xuejun Yuan, Weiren Yang, Faxiao Liu, Yanmeng Hou, Libo Huang, Shuzhen Jiang

**Affiliations:** ^1^Shandong Provincial Key Laboratory of Animal Biotechnology and Disease Control and Prevention, College of Animal Science and Veterinary Medicine, Shandong Agricultural University, Tai'an, China; ^2^College of Life and Sciences, Shandong Agricultural University, Tai'an, China

**Keywords:** zearalenone, gilts, ovarian development, oxidative stress, ovary

## Abstract

Zearalenone (ZEA) has an estrogen-like effect, which can injure the reproductive system of animals, causing infertility, and abortion in sows. However, the underlying mechanisms are still not clear. The objective of this study was to assess the effects of ZEA on the localization and expression of growth hormone (GH), growth hormone receptor (GHR), and heat shock protein 70 (Hsp70) in the ovaries of post-weaning gilts. Forty healthy post-weaning gilts were randomly provided one of four diets: normal basal diet supplemented with 0 (control), 0.5 (ZEA0.5), 1.0 (ZEA1.0), and 1.5 (ZEA1.5) mg ZEA/kg. Gilts were housed and fed individually for 35 days; the ovaries were collected after euthanasia for antioxidant index, relative mRNA and protein expression, and immunohistochemical analyses of GH, GHR, and Hsp70. The results revealed that the glutathione peroxidase and total superoxide dismutase levels decreased (*p* < 0.05), whereas the malondialdehyde level increased (*p* < 0.05) with increasing ZEA content. The localization pattern of GH, GHR, and Hsp70 in ZEA-treated gilts was the same as that in the control; however, the localization of yellow and brown immunoreactive substances of GH, GHR, and Hsp70 was stronger in the ZEA groups than in the control. The relative mRNA and protein expression of GHR and Hsp70 was the highest in the ZEA1.0 group (*p* < 0.05), whereas that of GH was the highest in the ZEA0.5 group (*p* < 0.05). The mRNA and protein expression of GH was lower in the ZEA1.5 group than in the control (*p* < 0.05). Hsp70 results showed adverse responses to increasing ZEA levels in gilt ovaries, suggesting that Hsp70 played an important role in alleviating ZEA-induced oxidative stress.

## Introduction

Zearalenone (ZEA; F-2 toxin), an estrogenic mycotoxin mainly produced by Fusarium fungi (1), is common in cereals, such as corn, barley, sorghum, rye, and wheat (2), and often found in processed products (3–5). ZEA is highly stable during storage and difficult to degrade via heating (6–9), posing health risks to animals and humans (10–12). ZEA can disturb the hormonal balance by binding to estrogen receptors, thereby inducing various diseases of the reproductive system (13, 14).

Our previous studies have shown that ZEA (1.05 mg ZEA/kg) induces deleterious effects on genital organs, serum hormones, and oxidative stress in post-weaning piglets in a gender-dependent manner (15, 16). ZEA at concentrations of 1.1–3.2 mg ZEA/kg linearly increases vulva size and genital organ weights, decreases the antioxidant capacity of the liver and serum (17), alters the structure of the uteri and ovaries, and promotes follicle growth by modulating the Wnt/β-catenin signaling pathway and estrogen-receptor gene expression in the ovaries of post-weaning gilts (18); however, the effect of ZEA on the distribution and expression of growth hormone (GH), GH receptor (GHR), and heat shock protein 70 (Hsp70) in the ovaries has not yet been fully elucidated. As the main hormone of the growth axis, GH controls ovarian follicular development, protects the antral follicle (19), accelerates the nuclear maturation of oocytes, and increases the number of primordial follicles (20). The GHR is a special GH-binding site. It was reported that GH binds to the GHR on the target cell membrane, transfers information to the cell, and fulfills its biological function (21). Several studies have reported the distribution or expression of GHR in the uteri of rats (22) and sheep (21) as well as in the ovaries of humans and chicken (23, 24); however, to date, no study has examined the expression of GHR in the ovaries of gilts challenged by ZEA.

Reportedly, ZEA induces oxidative stress by causing DNA damage, modulating cell-cycle progression, and triggering apoptosis and autophagy in mouse Leydig cells (25). As a highly conserved protein that is rapidly synthesized under stress, Hsp70 can reduce stress-induced damage to the body (26). Although a previous study showed that ZEA (0.5, 1.0, and 1.5 mg ZEA/kg) can enhance the distribution and mRNA expression of Hsp70 in post-weaning gilt uteri (27), little information is available on the effect of a low-dose ZEA (0.5–1.5 mg ZEA/kg) on Hsp70 localization and expression in post-weaning gilt ovaries.

Therefore, here, we aimed to explore the effects of ZEA on ovarian development and oxidative damage in post-weaning gilts through the localization and expression of GH, GHR, and Hsp70.

## Materials and Methods

### Preparation of Zearalenone-Contaminated Diet

Purified ZEA was purchased from Fermentek (Jerusalem, Israel). ZEA was dissolved in acetic ether and then poured onto talcum powder. The material was left overnight to allow the acetic ether to evaporate, and a ZEA premix of 1,000 mg ZEA/kg was subsequently prepared. This premix was then diluted with toxin-free corn meal to prepare a premix containing 10 mg ZEA/kg. The experimental diets were prepared in a bath and stored in separate covered containers before feeding. The doses of ZEA (0, 0.5, 1.0, and 1.5 mg ZEA/kg) used in the present study were based on the results reported by Jiang et al. (17) and Zhou et al. (28). The experimental diets were prepared a week before the trial. The toxin levels in the diet were examined immediately after sampling by the Qingdao Entry Exit Inspection and Quarantine Bureau before and at the end of the feeding experiment. The analyzed ZEA contents in the control, ZEA0.5, ZEA1.0, and ZEA1.5 groups were 0, 0.52 ± 0.07, 1.04 ± 0.03, and 1.51 ± 0.13 mg ZEA/kg, respectively, and no other mycotoxins were detected in any treatment diets.

### Experimental Design, Animals, and Management

The gilts used in all experiments were cared for in accordance with the guidelines for the care and use of laboratory animals prescribed by the Shandong Agricultural University Animal Care and Use Committee (Approval Number: # SDAUA-2019-019). Forty healthy post-weaning gilts (Duroc × Landrace × Large White) aged 35 days were selected for this study. The gilts were transferred to individual cages (0.48 m^2^) fitted with a plastic slatted floor, feed trough, and nipple drinker. The animals were then allocated to one of four dietary treatments (*n* = 10). The average body weight of the gilts was 14.01 ± 0.86 kg (mean ± standard deviation). The gilts were fed a basal diet according to the NRC (2012) supplemented with 0 (control), 0.5 (ZEA 0.5), 1.0 (ZEA1.0), or 1.5 (ZEA1.5) mg ZEA/kg for 35 days after 10 days of adaptation (Table 1). ZEA concentrations in the test diets were 0, 0.52 ± 0.07, 1.04 ± 0.03, and 1.51 ± 0.13 mg ZEA/kg, respectively. In all the treatment diets, no other mycotoxins were detected. Representative samples of feed were collected at the beginning and at the end of the experimental period for nutrient analyses according to the methods described by the AOAC (2012). The animal feeding experiment was performed at the Animal Nutrition Research Institute of Shandong Agricultural University, China. Before the test, the piggery was sterilized. During the first week of the experiment, the room temperature was maintained at 30°C; thereafter, it was maintained at 26–28°C. The relative humidity was ~65%.

**Table 1:** Ingredients and nutrient levels of the basal diet (air dry basis)^a^.

**Ingredients**	**Content (%)**	**Nutrients**^**c**^	
Corn	64.5	Digestible Energy, MJ/kg	13.81
Whey powder	5.0	Crude Protein (%)	19.82
Soybean meal	23.0	Calcium (%)	0.70
Fish meal	5.0	Total Phosphorus (%)	0.64
L-Lysine HCl	0.2	Lysine (%)	1.22
CaHPO_4_	0.7	Sulfur Amino Acid (%)	0.65
Pulverized Limestone	0.3	Threonine (%)	0.75
NaCl	0.3	Trptophan (%)	0.22
Premix^b^	1.0		
Total	100.0		

a *Treatments were basal diet supplemented with purified ZEA at the level of 0, 0.5, 1.0, or 1.5 mg kg^−1^, with analyzed ZEA concentrations of 0, 0.52 ± 0.07, 1.04 ± 0.03, and 1.51 ± 0.13 mg kg^−1^, respectively*.

b *Supplied per kg of diet: VA 3,300 IU, VD_3_ 330 IU, VE 24 IU, VK_3_ 0.75 mg, VB_1_ 1.50 mg, VB_2_ 5.25 mg, VB_12_ 0.026 mg, pantothenic acid 15.00 mg, niacin 22.50 mg, biotin 0.075 mg, folic acid 0.45 mg, Mn 6.00 mg, Fe 150 mg, Zn 150 mg, Cu 9.00 mg, I 0.21 mg, Se 0.45 mg*.

c *Digestible energy was the calculated value of the digestion test, and the other nutrient levels were analyzed value*.

### Sample Collection of Ovaries

Gilts were fasted for 12 h at the end of the feeding trial and then euthanized. The ovaries were immediately excised under sterile conditions. Ten ovaries (five right and five left ovaries) from five gilts per group were collected in an RNase-free 2-mL frozen tube, placed in liquid nitrogen, and stored at −80°C until subsequent analysis of the antioxidant index and relative expression of GH, GHR, and Hsp70. Another ten ovaries (five right and five left ovaries) from five gilts per group were promptly fixed in Bouin's solution for immunohistochemical analysis. Following fixation, 5-μm-thick sections were cut using a Leica RM 2,235 microtome (Lecia, Germany), mounted on poly L-lysine-coated glass slides, and dried overnight at 37°C before routine staining for immunohistochemical analysis.

### Determination of Antioxidant Enzyme Activity and Malondialdehyde Concentration

Ovarian tissues were thawed, washed with pre-cooled deionized water, and dried using a filter paper. Next, the tissues were mixed with physiological saline at a ratio of 1:9 (m/v) in an ice batch, moderately homogenized for 5 min, and then centrifuged at 1,500 × g for 10 min at 4°C. The supernatant was pipetted for the determination of the glutathione peroxidase (GSH-Px) (A005; Nanjing Jiancheng Bioengineering Institute, Jiangsu, China), total superoxide dismutase (TSOD) (A001-1-1; Nanjing Jiancheng Bioengineering Institute), and malondialdehyde (MDA) (A003-1; Nanjing Jiancheng Bioengineering Institute) levels according to the manufacturer's instructions.

### Total RNA Extraction, cDNA Preparation, and Quantitative Reverse Transcription Polymerase Chain Reaction (qRT-PCR)

According to the GH, GHR, Hsp70, and GAPDH gene sequences of pigs reported by GenBank, the corresponding primers were designed using Primer 6.0 and then synthesized by Bgi (Bgi Genomics Co., Ltd., China). Total RNA was extracted from the gilt ovaries using RNAiso Plus (Applied TaKaRa, China, Table 2) according to the manufacturer's instructions. An Eppendorf Biophotometer (RS323C; Eppendorf, Leipzig, Germany) was used to assess RNA purity and concentration at an absorbance ratio of 260/280 nm (values ranging from 1.8 to 2.0 indicated a pure RNA sample). Total RNA was reverse-transcribed into cDNA using a Reverse Transcription System kit (PrimeScript RT Master Mix, RR036A; Applied TaKaRa).

**Table 2:** Primer sequences of glycerol triphosphate dehydrogenase (GAPDH), growth hormone (GH), growth hormone receptor (GHR), and heat shock protein 70 (Hsp70).

**Target gene**	**Accession**	**Primer sequence (5^**′**^ to 3^**′**^)**	**Product size bp**
GAPDH	NM-001206359.1	F: ATGGTGAAGGTCGGAGTGAA	154
		R: CGTGGGTGGAATCATACTGG	
Hsp70	NM-001123127.1	F: GAGGTGGAGAGGATGGTT	292
		R: AGGCCTGGAGAAGATGG	
GH	M27326	F: AGAGGTACTCCATCCAGAAC	228
		R: GGTATGTCTCAGCCTTGTG	
GHR	DQ106869.1	F: CCTCAACTGGACTCTACTG	405
		R: ACACGCACTTCATACTCTT	

The total volume of the reaction mixture for quantitative PCR was 20 μL; it contained SYBR Premix Ex Taq-TIi RNaseH Plus (code: RR420A, Lot: AK7502; Applied TaKaRa). The optimized qRT-PCR protocol included an initial denaturation step at 95°C for 30 s, followed by 43 cycles at 95°C for 5 s, 60°C for 34 s, 95°C for 15 s, and 60°C for 60 s, with a final step at 95°C for 5 s. Further, qRT-PCR was performed on an AB7500 real-time PCR system (Applied Biosystems, CA, USA). The relative amounts of GH, GHR, and Hsp70 mRNA were expressed and calculated as being equal to 2-ΔΔCT (29).

### Immunohistochemical Analysis

Sections were processed according to the standard immunohistochemistry protocols, dewaxed, and rehydrated. Antigen retrieval was performed in a sodium citrate buffer (0.01 mol/L, pH 6.0) using a microwave unit for 20 min at full power. The sections were then washed with phosphate-buffered saline (PBS) (0.01 mol/L, pH 7.2). These sections were incubated in 10% hydrogen peroxide (H2O2) for 1.5 h and then incubated for 1 h in 10% normal goat serum (ZSGB-BIO, Beijing, China) to block non-specific binding. Immunohistochemical analysis was conducted according to the kit instructions (Polink-2 plus polymer HRP detection system for rabbit or mouse primary antibody, PV-9001/PV-9002; ZSGB-BIO). After washing with PBS, the sections were incubated overnight at 4°C with rabbit antibody GH (1:200, bs-6579R; BIOSS, Beijing, China), rabbit antibody GHR (1:200, bs-10661R; BIOSS), and monoclonal mouse antibody Hsp70 (1:200, BM0368; BOSTER, Wuhan, China). Then, the sections were washed with PBS; they were subsequently incubated in polymer helper at 37°C for 50 min and then with Polink-2 plus HRP anti-rabbit/mouse at 37°C for 1 h. After incubation, the sections were washed with PBS, immersed in diaminobenzidine tetrachloride (DAB kit; TIANGEN PA110, Beijing, China) for 1–3 min, counterstained with hematoxylin, and color-developed in tap water. The sections were then dehydrated, sealed in clear resin, mounted, and observed microscopically for the distribution of positive cells using a bright field of view.

### Immunohistochemical Analysis of GH, GHR, and Hsp70 Using Integrated Optical Density (IOD)

The histological sections of the ovaries were observed under a microscope (ELIPSE 80i; Nikon, Tokyo, Japan). Five stained sections randomly selected from ten gilts in each group were examined. To evaluate the amount of cell staining, the images were analyzed using an image analysis software (Image Pro-Plus 6.0; Media Cybernetics, Silver Spring, MD, USA). To assess the expression intensity of positive substance, we used an image analysis software (Image Pro-Plus 6.0; Media Cybernetics) to obtain the total cross-sectional IOD.

### Western Blot Analysis

Total protein was extracted from the ovarian tissue using a lysate containing phenylmethanesulfonyl fluoride [PMSF] (Beyotime, Shanghai, China) according to the manufacturer's instructions and detected using a BCA protein assay kit (Tiangen Biotech, China). The sample amount was 50–55 μg of protein per sample. Samples were separated by electrophoresis on polyacrylamide gels and subsequently transferred to nitrocellulose membranes. The membranes were incubated in 5% skimmed milk powder for 1.5 h, washed three times with Tris-buffered saline Tween-20 (TBST) (pH 7.6), and incubated with the primary antibodies [monoclonal anti-actin (1:1,000; Beyotime), rabbit antibody GH (1:300, bs-6579R; BIOSS), rabbit antibody GHR (1:300, bs-10661R; BIOSS), and monoclonal mouse antibody Hsp70 (1:300 BOSTER)] in primary antibody dilution buffer (Beyotime) at 4°C overnight. After washing with TBST, the membranes were incubated with anti-rabbit/mouse IgG antibody (1:2,000; CWBIO, Beijing, China) for 2 h at 37°C, followed by washing with TBST. Next, the membranes were immersed in a high-sensitivity luminescence reagent (BeyoECL Plus; Beyotime), exposed to film using a FusionCapt Advance FX7 (Beijing Oriental Science and Technology Development Co., Ltd., Beijing, China), and analyzed using Ipp 6.0 (Image Pro-Plus 6.0; Media Cybernetics).

### Statistical and Analysis

All data were analyzed using the general linear model procedure of SAS 9.2 (SAS Institute Inc., Cary, NC, USA) and one-way analysis of variance (ANOVA). Orthogonal polynomial contrasts were then used to determine linear responses to dietary ZEA concentrations. Significant differences between groups were analyzed using Duncan's multiple range tests. Statistical significance was considered at *p* < 0.05.

## Results

### Ovary Development

**Figure 1:**
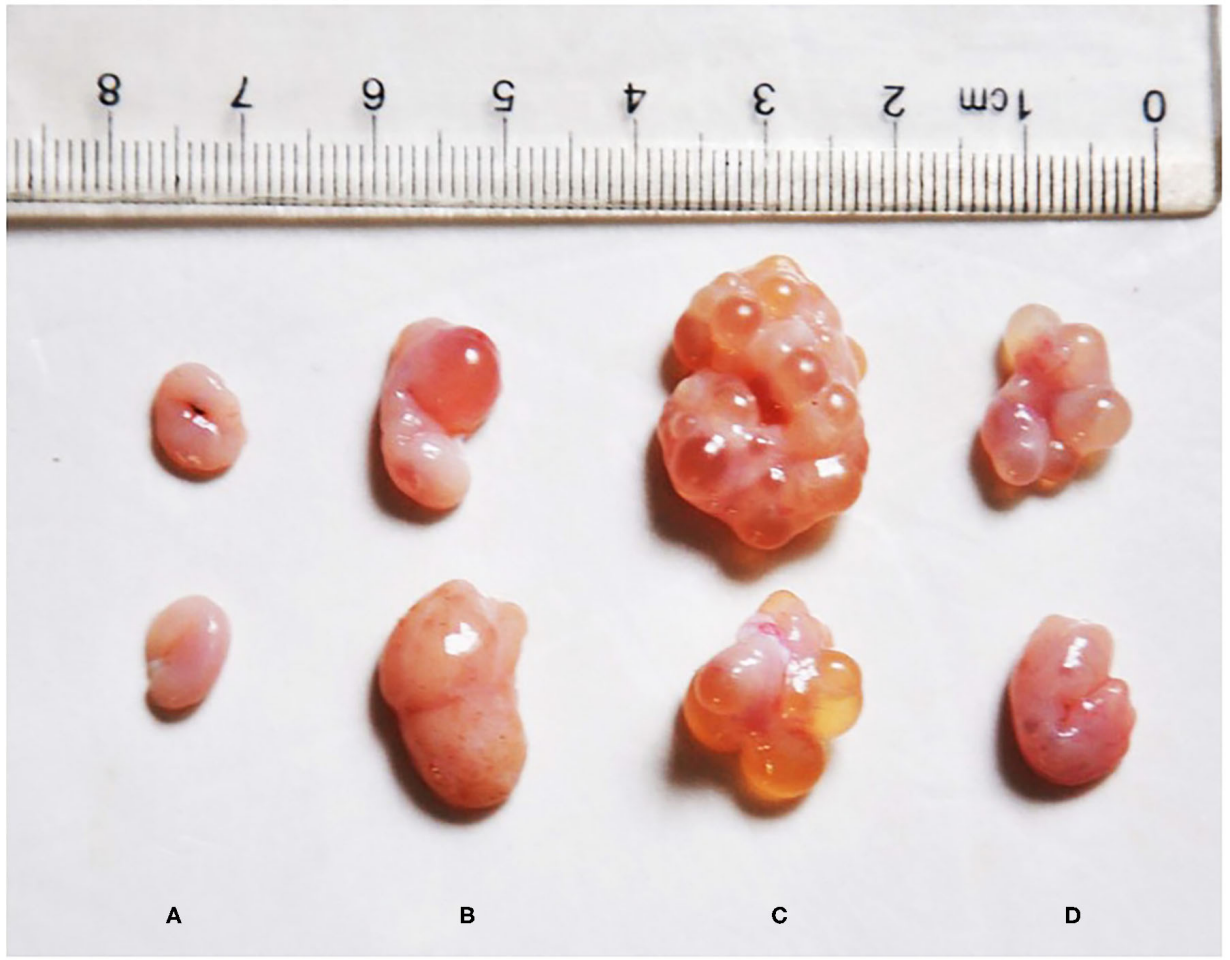
Effect of zearalenone (ZEA) on ovary in the post-weaning gilts. **(A–D)** Represent the basal diet with an addition of 0 (Control), 0.5 (ZEA0.5), 1.0 (ZEA1.0), and 1.5 (ZEA1.5) mg kg^−1^ ZEA, and with analyzed ZEA concentrations of 0, 0.52 ± 0.07, 1.04 ± 0.03, and 1.51 ± 0.13 mg kg^−1^, respectively.

Visually, the ovaries of the post-weaning gilts in the ZEA groups were larger than those in the control group (Figure 1). Moreover, some large follicles protruding from the surface of the ovary were observed in the ZEA groups. Macroscopic primary growth follicles were observed in the ZEA0.5 group, and a large number of secondary growth follicles were observed in the ZEA1.0 group; follicular atresia was observed in the ZEA1.5 group. Compared with the other groups, the ovarian weight was heaviest in the ZEA1.0 group (Table 3); the ovarian weight of the ZEA1.5 group was significantly higher than that of the ZEA0.5 group and the control (*p* < 0.05), which was consistent with the visual results.

**Table 3:** Effect of zearalenone (ZEA) on the ovarian weight, antioxidant enzymatic activity, and the malondialdehyde concentration in the ovary of post-weaning gilts.

**Items**	**Control**	**ZEA0.5**	**ZEA1.0**	**ZEA1.5**	**SEM**	***P*****-values**	
						**Treatment**	**Linear**
Ovarian Weight, g	0.637^c^	0.701^c^	2.262^a^	1.604^b^	0.015	<0.001	<0.001
GSH-Px, U·mg^−1^ pt.	217.32^a^	187.21^b^	141.14^c^	133.27^c^	1.876	<0.001	<0.001
TSOD, U·mg^−1^ pt.	103.45^a^	99.86^ab^	88.25^bc^	79.81^c^	0.703	<0.001	<0.001
MDA, nmol·mg^−1^ pt.	8.16^c^	9.92^bc^	10.77^b^	12.96^a^	0.171	<0.001	<0.001

*GSH-Px, Glutathione peroxidase; TSOD, Total superoxide dismutase; MDA, Malondialdehyde. Control, ZEA0.5, ZEA1.0, and ZEA1.5 represent the control diet with an addition of 0, 0.5, 1.0, and 1.5 mg kg^−1^ ZEA, and with analyzed ZEA concentrations of 0, 0.52 ± 0.07, 1.04 ± 0.03, and 1.51 ± 0.13 mg kg^−1^, respectively. Data per piglet were run in triplicate in a single assay to avoid inter-assay variation. SEM, standard error of the means. Values with a row with the different letters mean significantly different (P < 0.05)*.

### Antioxidant Enzymatic Activity and Malondialdehyde Concentration

The antioxidant index of the ovarian tissues showed that the GSH-Px and TSOD levels decreased linearly (*p* < 0.05, Table 3) with increasing ZEA levels. The level of GSH-Px in the control group was significantly higher than that in the ZEA groups (*p* < 0.05); moreover, the level of GSH-Px in the ZEA0.5 group was higher than that in the ZEA1.0 and ZEA1.5 groups (*p* < 0.05). The level of TSOD in the control group was higher than that in the ZEA1.0 and ZEA1.5 groups (*p* < 0.05), and it in the ZEA0.5 group was higher than that in the ZEA1.5 group (*p* < 0.05). However, the concentration of MDA increased linearly (*p* < 0.05) as the ZEA concentration in the diet increased. The level of MDA in the ZEA1.5 group was significantly higher than that in other groups (*p* < 0.05); further, the MDA in the ZEA1.0 group was higher than that in the control (*p* < 0.05).

### Immunohistochemical Staining Results

**Figure 2:**
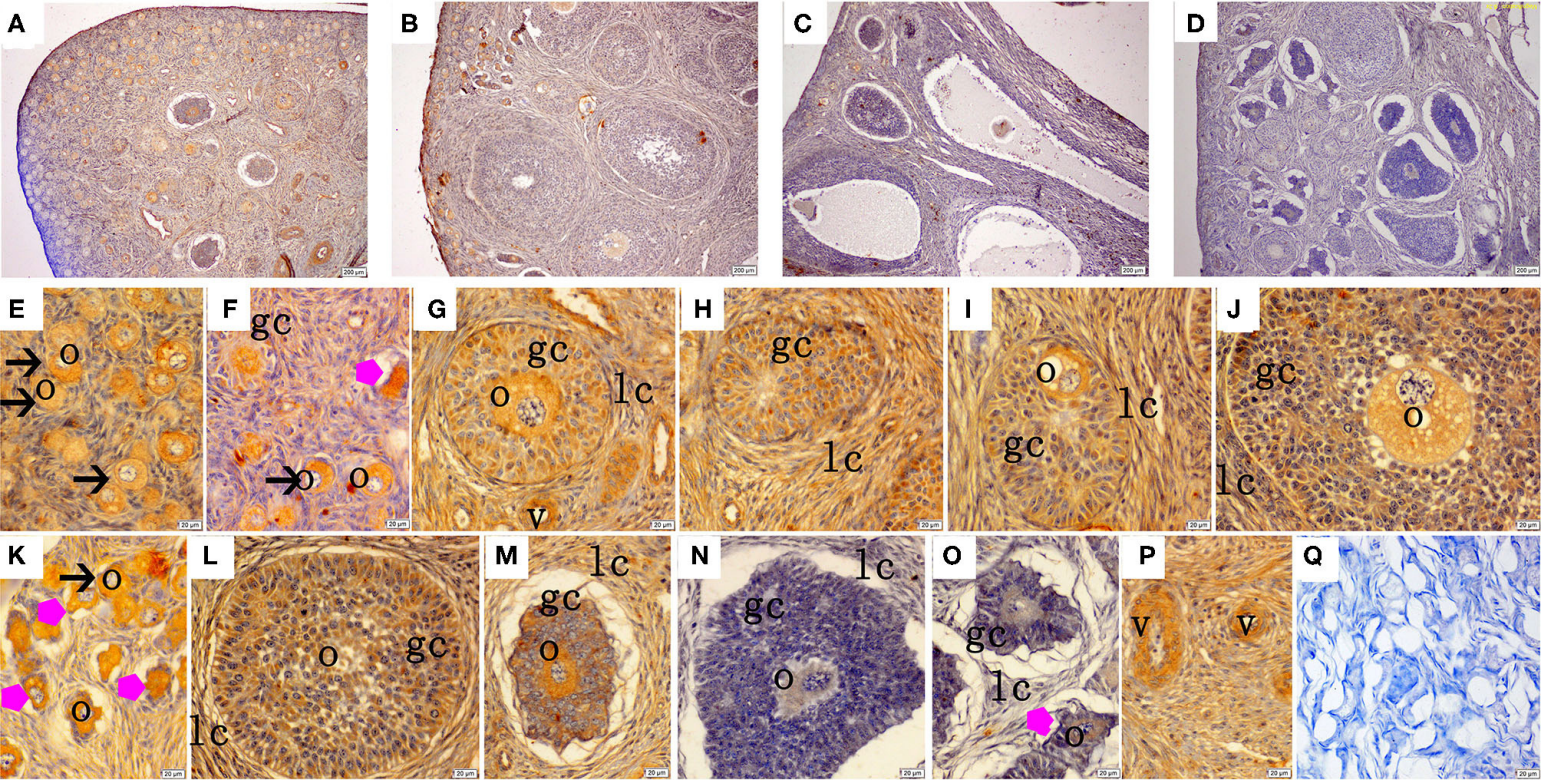
Effect of zearalenone (ZEA) on the growth hormone (GH) distribution in the ovary of post-weaning gilts. **(A–D)** Represent the basal diet with an addition of 0 (Control), 0.5 (ZEA0.5), 1.0 (ZEA1.0), and 1.5 (ZEA1.5) mg kg^−1^ ZEA, and with analyzed ZEA concentrations of 0, 0.52 ± 0.07, 1.04 ± 0.03, and 1.51 ± 0.13 mg kg^−1^, respectively. The **(E)** was the primordial follicles of the Control. **(F–J)** Was the healthy growing follicle of the ZEA-treatment. **(K)** Was the atretic primordial and growing follicle of the ZEA-treatment. **(L,M)** Was the atretic growing follicle of the Control. **(N,O)** Was the atretic growing follicle of the ZEA1.5 treatment. **(E–M)** Indicates the distribution of positive substances on the oocyte and granulosa cell. **(P)** Indicates the distribution of positive substances on the vascular smooth muscle. The straight arrow (black) indicate the healthy primordial follicle **(E,K)**, and the star (purple) indicate the atresia of the primordial follicle **(K)**. The star (purple) of O indicate the atresia growing follicle. The o, gc, lc, and v represent oocyte, granulosa cell, theca cell, and vessel, respectively. The scale was 100 μm **(A–D,P)** and 20 μm **(E–P)**, respectively. **(Q)** oocyte.

**Figure 3:**
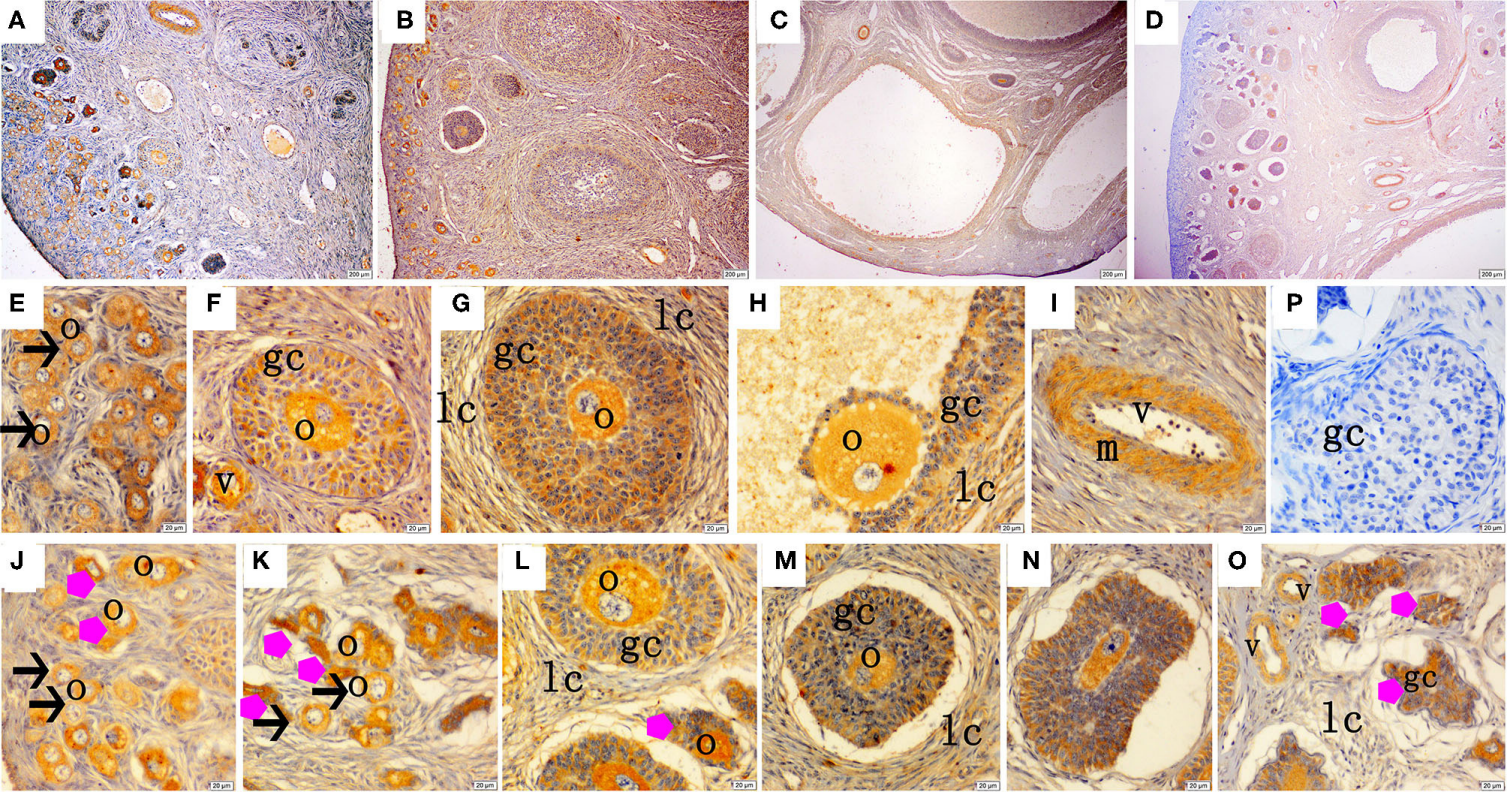
Effect of zearalenone (ZEA) on the growth hormone receptor (GHR) distribution in the ovary of post-weaning gilts. **(A–D)** Represent the basal diet with an addition of 0 (Control), 0.5 (ZEA0.5), 1.0 (ZEA1.0), and 1.5 (ZEA1.5) mg kg^−1^ ZEA, and with analyzed ZEA concentrations of 0, 0.52 ± 0.07, 1.04 ± 0.03, and 1.51 ± 0.13 mg kg^−1^, respectively. The **(E)** was the primordial follicles of the Control. **(F–H)** Was the healthy growing follicle of the ZEA-treatment. **(J)** Was the atretic growing follicle of the ZEA0.5-treatment. **(K,O)** Was the atretic growing follicle of the ZEA1.5-treatment. **(L–N)** Was the atretic growing follicle of the ZEA0.5, ZEA 1.0-treatment. The straight arrow (black) indicate the healthy primordial follicle **(E)**, and the star (purple) indicate the atresia primordial follicle **(J,K)**. The star (purple) of O indicate the atresia growing follicle. The o, gc, and lc represent the oocyte, granulosa cell, and theca cell, respectively, v and m represent the vessel and the vascular smooth muscle, respectively. The scale was 100 μm **(A–D)** and 20 μm **(E–O)**, respectively.

**Figure 4:**
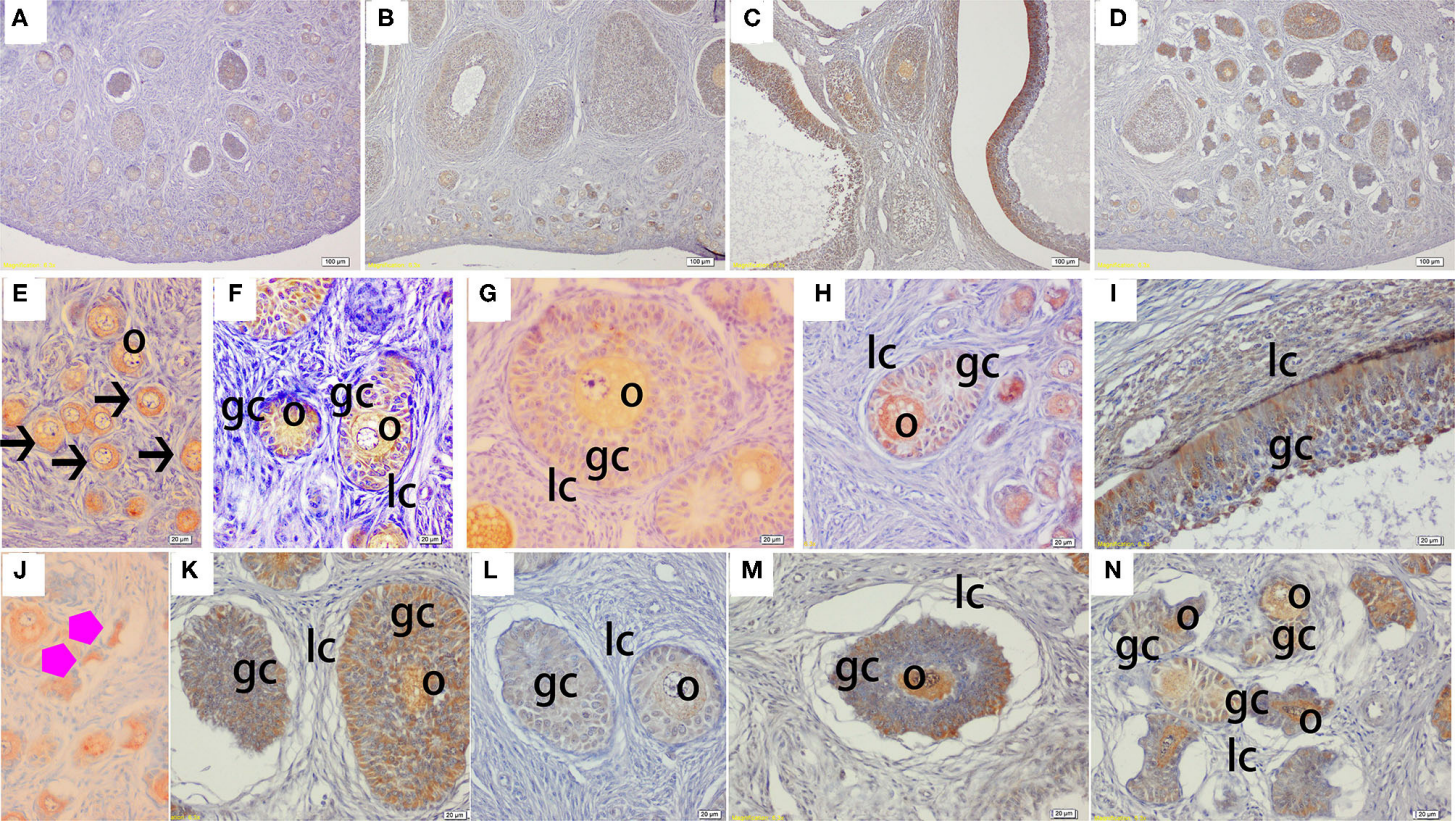
Effect of zearalenone (ZEA) on the heat shock protein 70 (Hsp70) distribution in the ovary of post-weaning gilts. **(A–D)** Represent the basal diet with an addition of 0 (Control), 0.5 (ZEA0.5), 1.0 (ZEA1.0), and 1.5 (ZEA1.5) mg kg^−1^ ZEA, and with analyzed ZEA concentrations of 0, 0.52 ± 0.07, 1.04 ± 0.03, and 1.51 ± 0.13 mg kg^−1^, respectively. The **(E)** was the primordial follicles of the Control. **(F–I)** Was the healthy growing follicle of the ZEA-treatment. **(J)** Was the atretic primordial follicles of the ZEA0.5-treatment. **(K–N)** Indicate the atretic growing follicles of the ZEA-treatment. The straight arrow (black) indicate the healthy primordial follicle **(E)**, and the star (purple) indicate the atresia primordial follicle **(J)**. The o, gc, and lc represent the oocyte, granulosa cell, and theca cell, respectively. The scale was 100 μm **(A–D)** and 20 μm **(E–N)**, respectively.

The immunohistochemical staining results revealed that GH and GHR immunoreactive substances were mainly localized in the cytoplasm of the oocytes, granulosa cells (GCs), and vessel endothelial cells of the ovaries of the gilts (Figures 2, 3). The level of GH in the oocytes of healthy primordial follicles (Figure 2E) and primary growth follicles (Figures 2F,G) with fewer GC layers was higher than that in the oocytes of growth follicles with more GC layers (Figures 2I,J). Moreover, the level of GH in the oocytes and GCs of atresia growth follicles gradually decreased with an increasing degree of atresia. The highest GHR level was observed in the oocytes of healthy primordial follicles (Figure 3E). With the development of the follicles, the oocytes in the growing follicles gradually decreased (Figures 3G,H), whereas the GCs increased. Interestingly, the oocytes and GCs of atresia follicles did not appear to decrease as a result of follicular atresia (Figures 3K–O). Hsp70 was mainly localized in the cytoplasm of the oocytes and GCs in the ovaries of the gilts (Figure 4). The larger the follicle, the higher the number of GCs with positive substances (Figures 4G–I). Hsp70 expression in the oocytes was higher in atresia follicles than in healthy growing follicles (Figure 4J). With the enhancement of atresia, the GCs gradually decreased and the oocytes increased (Figures 4K,M,N).

The IOD of GH, GHR, and Hsp70 in the ovaries of the gilts showed consistent results with the above immunochemical analysis results (Table 4). The IOD of GH and Hsp70 showed a linear (*p* < 0.05) increase with increasing levels of ZEA. The IOD of GH in the ZEA0.5 group was higher (*p* < 0.05) than that in the ZEA1.5 group. The IOD of GHR in the ZEA1.0 group was higher than that in the ZEA0.5 group (*p* < 0.05); further, the IOD of GHR in the ZEA0.5 group was higher than that in the ZEA1.5 and control groups (*p* < 0.05). The IOD of Hsp70 in the ZEA1.0 group was higher than that in the ZEA0.5 and ZEA1.5 groups (*p* < 0.05); further, the IOD of Hsp70 in the ZEA0.5 and ZEA1.5 groups was higher than that in the control group (*p* < 0.05).

**Table 4:** Effects of zearalenone (ZEA) on the mRNA and protein relative expression, and the integrated optical density (IOD) of growth hormone (GH), growth hormone receptor (GHR), and heat shock protein 70 (Hsp70) in the ovary of post-weaning gilts.

**Items**	**Control**	**ZEA0.5**	**ZEA1.0**	**ZEA1.5**	**SEM**	***P*****-values**	
						**Treatment**	**Linear**
**mRNA expression**							
GH	1.13^b^	1.77^a^	1.10^b^	0.55^c^	0.116	<0.001	0.019
GHR	0.63^c^	1.39^b^	1.67^a^	0.74^c^	0.098	<0.001	0.492
Hsp70	0.72^b^	1.01^b^	1.98^a^	0.95^b^	0.123	<0.001	0.133
**Protein expression**							
GH	1.00^b^	1.77^a^	0.97^b^	0.56^c^	0.135	<0.001	0.080
GHR	1.00^c^	1.68^b^	2.11^a^	1.24^c^	0.132	<0.001	0.357
Hsp70	1.00^d^	1.74^b^	2.11^a^	1.38^c^	0.130	<0.001	0.209
**IOD (×10**^**3**^**)**							
GH	27.32^ab^	29.91^a^	27.45^ab^	24.28^b^	0.638	0.016	0.002
GHR	25.63^c^	40.63^b^	48.05^a^	27.02^c^	1.590	<0.001	0.422
Hsp70	19.93^c^	30.96^b^	40.04^a^	30.55^b^	1.126	<0.001	<0.001

*Control, ZEA0.5, ZEA1.0, and ZEA1.5 represent the control diet with an addition of 0, 0.5, 1.0, and 1.5 mg kg^−1^ ZEA, and with analyzed ZEA concentrations of 0, 0.52 ± 0.07, 1.04 ± 0.03, and 1.51 ± 0.13 mg kg^−1^, respectively. Data per piglet for mRNA and protein relative expression were run in triplicate in a single assay to avoid inter-assay variation. SEM, standard error of the means. Values with a row with different letters mean significantly different (P < 0.05)*.

### Relative mRNA Expression of GH, GHR, and Hsp70

The relative mRNA expression levels of GH, GHR, and Hsp70 were consistent with the immunohistochemical analysis results (Table 4). The relative mRNA expression of GH in the ovaries of the gilts decreased linearly (*p* < 0.05) as the ZEA concentration in the diet increased. The mRNA expression of GH in the ZEA0.5 group was higher (*p* < 0.05) than that in the ZEA1.0 and control groups; further, mRNA expression of GH in the ZEA1.0 and control groups was higher than that in the ZEA1.5 group (*p* < 0.05). The mRNA expression of GHR in the ZEA1.0 group was higher (*p* < 0.05) than that in the ZEA0.5 group; further, the mRNA expression of GHR in the ZEA0.5 group was higher than that in the ZEA1.5 and control groups (*p* < 0.05). The mRNA expression of Hsp70 in the ZEA0.5, ZEA1.5, and control groups was similar (*p* > 0.05) but lower than that in the ZEA1.0 group (*p* < 0.05).

### Protein Expression of GH, GHR, and Hsp70

**Figure 5:**
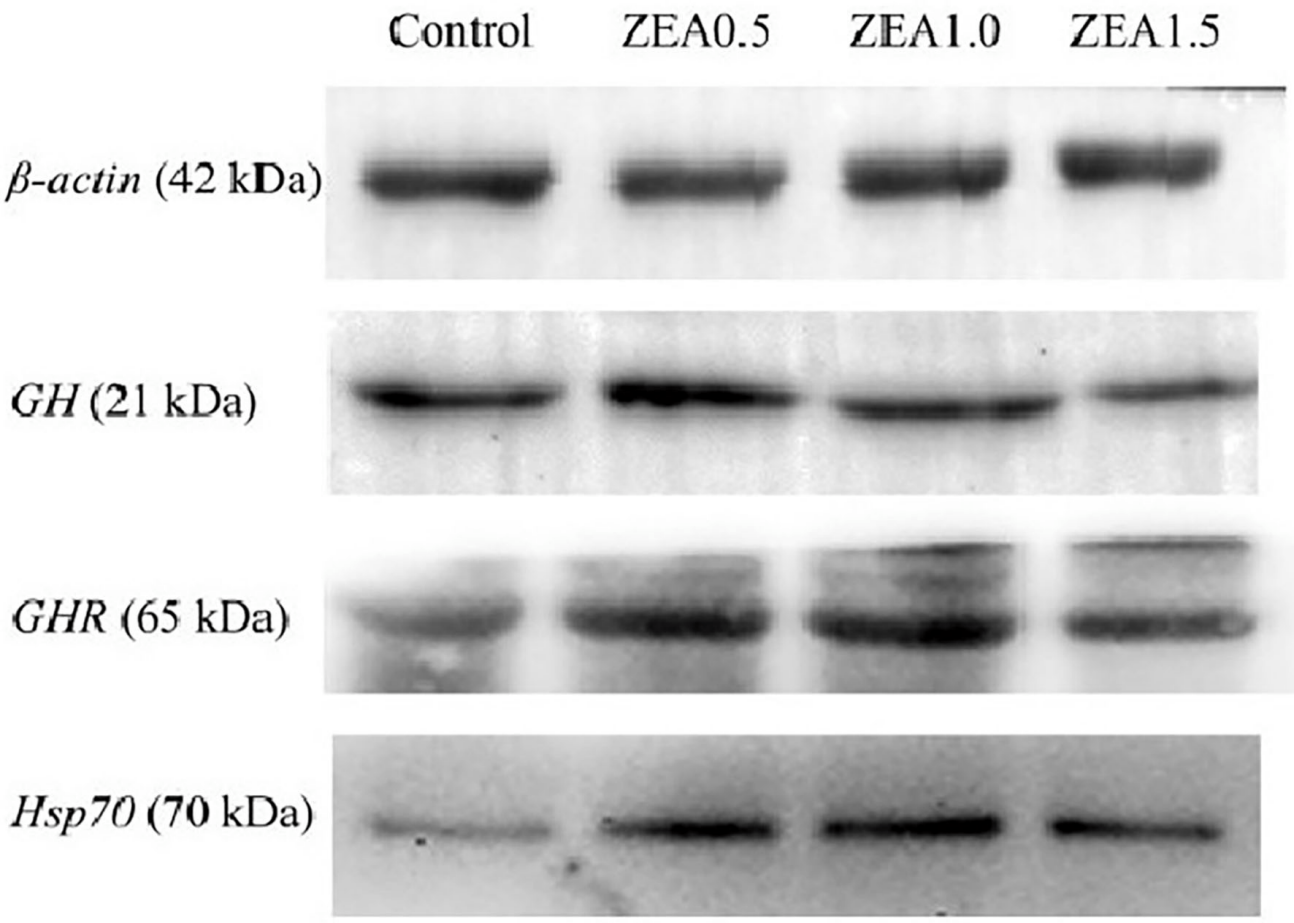
Western blot analysis of growth hormone (GH), growth hormone receptor (GHR), and heat shock protein 70 (Hsp70) in the ovary of post-weaning gilts. Control, ZEA0.5, ZEA1.0, and ZEA1.5 represent the control diet with an addition of 0, 0.5, 1.0, and 1.5 mg kg^−1^ ZEA, and with analyzed ZEA concentrations of 0, 0.52 ± 0.07, 1.04 ± 0.03, and 1.51 ± 0.13 mg kg^−1^, respectively.

Western blot analysis (Figure 5) revealed positive bands of appropriate sizes for all the studied genes (β-actin, GH, GHR, and Hsp70). The analysis results of protein expression were consistent with those of the mRNA expression (Table 4). The order of the relative protein expression in the ovaries of the gilts from all the groups is as follows: ZEA0.5 > ZEA1.0 = control > ZEA1.5 for GH (*p* > 0.05); ZEA1.0 > ZEA0.5 > ZEA1.5 = control for GHR (*p* > 0.05); and ZEA1.0 > ZEA0.5 > ZEA1.5 > control for Hsp70 (*p* > 0.05).

## Discussion

Our previous studies showed that it had similar daily gain, feed intake, and feed efficiency in the gilts of all the groups (30), which indicated that the gilts under treatment likely consumed a similar amount of digestible energy and other nutrients. ZEA (0.5–1.5 mg ZEA/kg) caused a reduction in the proportion of primordial follicles (18), which lead to a reduction in the layer thickness of the primordial follicle. At the same time, the ovarian index of post-weaning piglets increased linearly with an increase of ZEA in the diet, but the index in the ZEA1.5 group was lower than that in the ZEA1.0 group (31), which was consistent with the apparent ovarian development in the present study. Therefore, the above results suggested that ZEA could increase the number of atresia follicles while promoting ovarian development, which might cause ovarian atrophy.

### GH and GHR Expression in the Ovaries

ZEA has been reported to exert estrogenic activities, regulating the development of the reproductive system of female animals (32–34). We observed that ZEA upregulated the expression of GH at 0.5 mg ZEA/kg, and GHR at 0.5 and 1.0 mg ZEA/kg as well as downregulated the expression of GH at 1.5 mg ZEA/kg, compared with that in the control group, which is a significant finding in the current study. This finding is consistent with our previous results that 0.5 and 1.0 mg ZEA/kg promotes the development of primordial/primary follicles into secondary growth follicles and increases the diameter of secondary growth follicles (18, 35); however, we also observed a higher number of atresia growth follicles in the 1.5 mg ZEA/kg group than that in the 0.5 and 1.0 mg ZEA/kg groups.

GH and GHR are present in chicken ovary and primarily localized in the granulosa and theca layers (23, 36). Similarly, in the present study, GH and GHR were mainly localized in the cytoplasm of the oocytes and GCs in the ovaries of the gilts. In contrast, GHR mRNA expression was not detected in secondary and early tertiary caprine follicles (37). In addition, it is noteworthy that both GH and GHR showed yellow and brown staining in the vascular smooth muscle cells, suggesting that GH and GHR played roles in angiogenesis or blood flow regulation in the ovaries of gilts.

It has been reported that GH combines with ghrelin to promote various ovarian activities, such as steroidogenesis, ovarian puberty initiation, gonadotropin responsiveness, and ovulation (38). Studies have shown that GH stimulates cell proliferation and inhibits cell apoptosis in chicken and mammalian ovaries during sexual maturation (39, 40). In addition, Socha and Hrabia indicated that GH promotes the formation and development of prehierarchical follicles in hen ovary during a pause in egg-laying by regulating cell proliferation and apoptosis (41). Our previous studies indicated that ZEA upregulates the expression of GH and GHR in the uterus, which leads to vulva swelling and uterine hypertrophy in weaning gilts (42, 43). However, in the present study, we found that ZEA increased GH expression at 0.5 mg ZEA/kg and GHR expression at 0.5 and 1.0 mg ZEA/kg, and decreased GH expression at 1.5 mg ZEA/kg. Thus, it is suggested that these alterations in GH and GHR expression by ZEA were organ- and dose-dependent and that low-dose ZEA (0.5 and 1.0 mg ZEA/kg) exerted estrogenic effects by upregulating GH and GHR expression, promoting the development of ovaries and follicles, and leading to an increase in ovarian volume. However, the toxic effects of ZEA were more potent than its estrogenic effects, as 1.5 mg ZEA/kg caused a lower GH expression than 0.5 and 1.0 mg ZEA/kg. In short, the dose-effect relationship of ZEA was observed on the localization and expression of GH and GHR in gilt ovaries; however, the specific underlying molecular mechanism is still unclear and needs to be further studied *in vitro*.

### Antioxidant Index and Hsp70 Expression

Numerous studies have noted that ZEA exerts adverse effects on antioxidant activity both *in vivo* and *in vitro*. It has been reported that ZEA-induced oxidative stress is accompanied by an increase in oxygen free radicals, cell membrane rupture, and considerable damage to genomic DNA (44). ZEA may induce oxidative stress in various organs. Salah-Abbes et al. (45) reported that ZEA (40 mg ZEA/kg) significantly reduces SOD and GSH-Px activity in mouse testes. A diet supplemented with 1.1–3.2 mg ZEA/kg decreased the serum and liver TSOD and GSH-Px activities in both the serum and liver of piglets but increased the MDA concentration (17). According to the results reported by Yuan et al., ZEA at 60, 90, and 120 μmol/L decreases the activity of antioxidant enzymes (SOD and GSH-Px) and increases the concentration of MDA in ovarian granule cells (46). Moreover, our previous results indicated that ZEA decreases SOD and GSH-Px activity and increases MDA content in the ileum of piglets and inhibits intestinal damage by activating the Keap1-Nrf2 signaling pathway (47). Furthermore, ZEA (10, 20, 40 μmol/L) decreases cellular activities, inhibits protein and DNA production, increases the MDA content, damages the cell structure, and elicits the stress response (48). Antioxidant enzymes (such as SOD and GSH-Px) represent the primary defense system preventing organ injuries due to an excessive quantity of reactive oxygen species that cause cellular lipid peroxidation (49). Therefore, the decreased TSOD and GSH-Px activity and increased MDA content in the ovaries of the post-weaning gilts in the present study indicated that ZEA induced oxidative stress in the ovary, resulting in the disruption of ovary development. It has been reported that the decreased GSH-Px activity may be the result of the conjugation of GSH-Px with ZEA or its metabolites (50); further, the increased MDA content might be caused by the decreased TSOD and GSH-Px activity associated with ZEA supplementation. However, the molecular mechanism by which ZEA induced oxidative stress in the ovaries of the post-weaning gilts needs to be further investigated.

The Hsp family, which is highly conserved, has attracted considerable attention (51). Hsp70, the most important member of the Hsp family, improves cell tolerance to stressors, resists apoptosis, and reduces cell peroxidation and inflammatory injury (52). A study reported that the heat shock response is a key cellular response to xenobiotic exposure; moreover, it can potentially be used as an early marker of toxicity (53). Research has also shown that, under Hsp70 overexpression, intestinal epithelial cells were protected from hypoxia reoxygenation injury (54). Moreover, a study reported that elevated Hsp70 production after sub-LHS provides cytoprotection against ZEA cytotoxicity by diminishing oxidative damage (55). Notably, the relative expression of Hsp70 mRNA and protein in the present study did not show linear changes with increasing ZEA, which is inconsistent with our previous results: linear changes in uterine Hsp70 expression in weaning gilts (55). However, the expression of Hsp70 was consistent with that of GHR under ZEA treatment, which suggested that the oxidative stress induced by ZEA (0.5, 1.0, and 1.5 mg ZEA/kg) was closely related to ovarian development. With increasing ZEA concentrations, oxidative stress intensified, thereby causing irreversible damage to the ovaries, leading to a decline in GH expression and subsequent ovarian atrophy. Therefore, we think that the weight of ovary of zea1.5 treatment is lower than that treated with zea1.0 is closely related to the significantly decreased expression of GHR and GH. Nevertheless, the molecular mechanism underlying the association of Hsp70 with oxidative stress in the ovaries of post-weaning gilts under high-dose ZEA treatment will be our next focal research issue.

## Conclusions

In conclusion, low-dose ZEA at 0.5 and 1.0 mg ZEA/kg promoted ovarian development by upregulating GH and GHR expression. However, 1.5 mg ZEA/kg ZEA decreased the expression of GH and GHR, resulting in the lower ovarian weight of ZEA1.5 than that of ZEA1.0. In addition, the expression of HSP70 was consistent with that of GHR, which suggested that the ovarian development induced by ZEA (0.5, 1.0, and 1.5 mg/kg) may be closely related to oxidative stress. But further *in vitro* studies are needed to determine the relationship between oxidative stress and ovarian development in gilts challenged by ZEA.

## Data Availability Statement

The raw data supporting the conclusions of this article will be made available by the authors, without undue reservation.

## Ethics Statement

The gilts used in all experiments were cared for in accordance with the guidelines for Shandong Agricultural University Animal Care and Use Committee (Approval Number: # SDAUA-2019-019).

## Author Contributions

SJ and WY conceived this research and designed experiments. LH, XL, and YH participated in the design and interpretation of the date. TS, XY, and FL performed the experiments and analysis. TS, SJ, and LH wrote the paper and participated in the revisions of it. All authors read and approved the final manuscript.

## Conflict of Interest

The authors declare that the research was conducted in the absence of any commercial or financial relationships that could be construed as a potential conflict of interest.
